# Peroxisomal ABC Transporters: An Update

**DOI:** 10.3390/ijms22116093

**Published:** 2021-06-05

**Authors:** Ali Tawbeh, Catherine Gondcaille, Doriane Trompier, Stéphane Savary

**Affiliations:** Laboratoire Bio-PeroxIL EA7270, University of Bourgogne Franche-Comté, 6 Boulevard Gabriel, 21000 Dijon, France; ali.tawbeh@u-bourgogne.fr (A.T.); Catherine.Gondcaille@u-bourgogne.fr (C.G.); doriane.trompier@u-bourgogne.fr (D.T.)

**Keywords:** ABC transporters, peroxisome, adrenoleukodystrophy, fatty acids

## Abstract

ATP-binding cassette (ABC) transporters constitute one of the largest superfamilies of conserved proteins from bacteria to mammals. In humans, three members of this family are expressed in the peroxisomal membrane and belong to the subfamily D: ABCD1 (ALDP), ABCD2 (ALDRP), and ABCD3 (PMP70). These half-transporters must dimerize to form a functional transporter, but they are thought to exist primarily as tetramers. They possess overlapping but specific substrate specificity, allowing the transport of various lipids into the peroxisomal matrix. The defects of ABCD1 and ABCD3 are responsible for two genetic disorders called X-linked adrenoleukodystrophy and congenital bile acid synthesis defect 5, respectively. In addition to their role in peroxisome metabolism, it has recently been proposed that peroxisomal ABC transporters participate in cell signaling and cell control, particularly in cancer. This review presents an overview of the knowledge on the structure, function, and mechanisms involving these proteins and their link to pathologies. We summarize the different in vitro and in vivo models existing across the species to study peroxisomal ABC transporters and the consequences of their defects. Finally, an overview of the known and possible interactome involving these proteins, which reveal putative and unexpected new functions, is shown and discussed.

## 1. Introduction

ATP-binding cassette (ABC) transporters constitute a superfamily of membrane transporter proteins that actively translocate a wide range of molecules, from simple molecules (fatty acids (FAs), sugars, nucleosides, and amino acids) to complex organic compounds (lipids, oligonucleotides, polysaccharides, and proteins) [1]. Transport of substrates is dependent on the hydrolysis of ATP, which releases energy that can be used to accumulate substances in the cellular compartments or export them to the outside. ABC transporters are distributed not only in the plasma membrane of both prokaryotes and eukaryotes, but also in the membranes of the organelles of eukaryotic cells such as peroxisomes, mitochondria, lysosomes, and endoplasmic reticulum (ER). Based on their amino acid homology and structural configuration, ABC transporters in humans are classified into seven subfamilies, A to G, comprising a total of 48 ABC transporters, many of which are implicated in diseases [2]. ABC transporters of subfamily D include four proteins in mammals: ABCD1 [adrenoleukodystrophy protein (ALDP)], ABCD2 [adrenoleukodystrophy-related protein (ALDRP)], ABCD3 [70 kDa peroxisomal membrane protein (PMP70)], and ABCD4 [peroxisomal membrane protein 69 (PMP69)] [3]. ABCD1, ABCD2, and ABCD3 are located in the peroxisomal membrane. ABCD4 was identified by homology search for ALDP and PMP70 related sequences in the database of expressed sequence tags, and was initially considered peroxisomal despite the absence of a membrane peroxisomal targeting signal [4]. More recently, several studies have demonstrated that ABCD4 resides in the endoplasmic reticulum and lysosomes, and that its function is associated with cobalamin metabolism [3,5,6].

**Figure 1:**
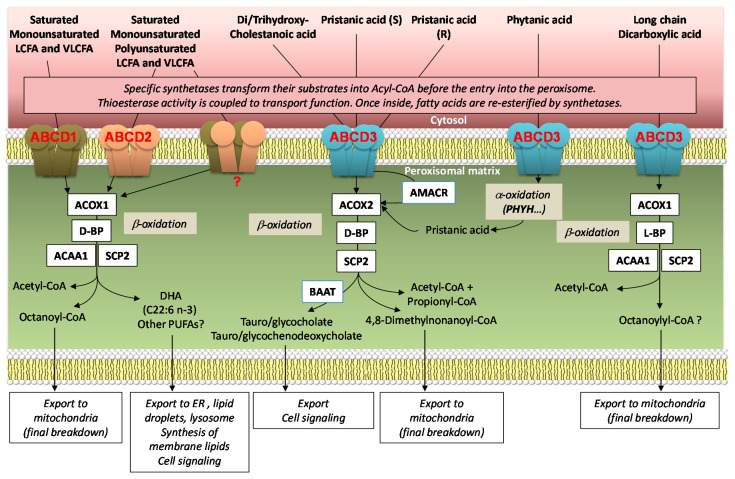
Peroxisomal ABC transporters and their involvement in lipid metabolism. Peroxisomal ABC transporters are represented as homo or heterotetramers with their preferential substrates and their involvement in metabolic routes, including several enzymatic steps, catalyzed by acyl-CoA oxidase 1 and 2 (ACOX1 and ACOX2), D- and L-bifunctional protein (D-BP and L-BP), acetyl-CoA Acyltransferase 1 (3-ketoacyl-CoA thiolase, ACAA1), sterol carrier protein 2 (SCPX thiolase, SCP2), alpha-methylacyl-CoA racemase (AMACR), bile acid-CoA:amino acid N-acyltransferase (BAAT), and phytanoyl-CoA hydroxylase (PHYH).

The three human peroxisomal ABC transporters play an important role in the transport of various lipid substrates into the peroxisome for their shortening by β-oxidation (Figure 1). β-oxidation of FAs is a conserved process of peroxisomes by which acyl groups are degraded two carbons at a time after being activated to form the corresponding CoA derivative by a specific acyl-CoA synthetase located at the peroxisomal membrane [7]. The β-oxidation process exists in mitochondria for medium- and long-chain fatty acids (MCFAs and LCFAs) and is necessary to terminate degradation of octanoyl-CoA coming from peroxisomes. However, very long-chain fatty acids (VLCFAs, number of carbon atoms >22) are exclusively β-oxidized into the peroxisome, and this organelle is therefore essential, especially in the brain [8]. Moreover, polyunsaturated fatty acid (PUFA) synthesis may require a peroxisomal cycle of β-oxidation, as in the case of docosahexaenoic acid (DHA, C22:6 n-3) synthesis from its precursor (C24:6 n-3) [9]. It is important to note that DHA is not only of great value by itself as a component of cell membranes, but is also the source of eicosanoids associated with several key signaling functions [10]. 

Thus, peroxisomal β-oxidation may not be considered a simple catabolic process of fatty acids. The role of peroxisomal ABC transporters is therefore not restricted to the catabolic function of peroxisomes, but is fully associated with their various metabolic functions including synthesis and degradation of lipids, cell signaling, inflammation control, and redox homeostasis [11,12,13,14,15].

## 2. Structure, Function, and Mechanism of Transport 

### 2.1. Structure

**Figure 2:**
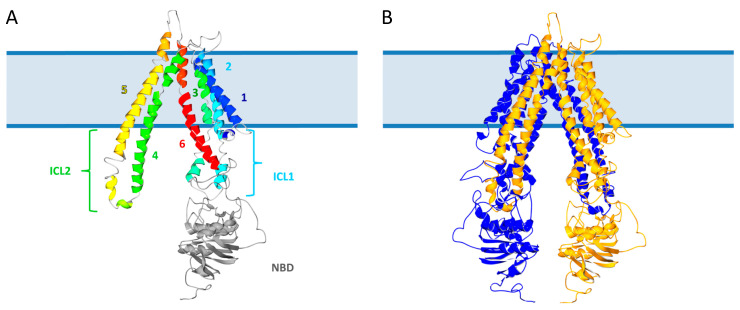
Structural model of human ABCD1 (reprinted from [17]). (**A**) Ribbon representation of the ABCD1 monomer. TMD helices are numbered from 1 to 6 and rainbow colored from dark blue to red. NBD is in light grey, and intracellular loops (ICL) 1 and 2 are indicated. (**B**) Ribbon representation of the ABCD1 homodimer with the two subunits respectively colored in dark blue and yellow.

The general structure of eukaryotic ABC transporters is a four functional unit organization, comprising two transmembrane domains (TMDs) and two nucleotide binding domains (NBDs). NBDs bind and hydrolyze ATP to trigger conformational changes in the TMDs, resulting in unidirectional transport across the membrane [1]. Human peroxisomal ABC transporters have a half-transporter structure, with only one TMD and one NBD [16]. In 2017, we proposed a structural model of human ABCD1 based on the crystal structure of the mitochondrial ABC transporter ABCB10, which shows not only the putative structure of ABCD1 in a membrane context but also the complex intricacy of α-helices that constitute the whole transmembrane domain (Figure 2) [17]. Therefore, peroxisomal ABC half-transporters need to homo- or heterodimerize in the peroxisomal membrane in order to constitute a full, active transporter [18,19].

Data shows that ABCD1, ABCD2, and ABCD3 are able to interact as homodimers or heterodimers [20,21,22], although both ABCD1 and ABCD3 are mainly found as homodimers in mammalian peroxisomal membranes [23,24,25]. Moreover, ABCD1 and ABCD2 homodimers are functional [26,27]. However, the fact that nonfunctional ABCD2 has a transdominant negative effect on ABCD1 [20] suggests that heterodimers of ABCD1 and ABCD2 are also functional and can exist within cells and tissues expressing both proteins. Besides, chimeric proteins consisting of homo- and heterodimers of ABCD1 and ABCD2 are functionally active [19]. Concerning ABCD3, although homodimers and heterodimers with ABCD1 and ABCD2 have been described [22,23,25,28,29], no data is available about the functional value of the ABCD3 dimers. Surprisingly, ABCD1 and ABCD3 were found in different detergent-resistant microdomains [29], implying that these proteins have a different environment in the peroxisomal lipid bilayer, questioning the biological relevance of the ABCD1 and ABCD3 heterodimers. Additionally, native PAGE experiments concerning complex oligomerization confirm that ABCD1 and ABCD2 exist predominantly as homo-tetramers, although both homo- and hetero-tetramers are present [28]. Therefore, we cannot rule out the possibility that hetero-interaction between ABCD1 and ABCD2 occurs in hetero-tetramers composed of two distinct homodimers rather than in complexes composed of two heterodimers. Finally, it remains unclear whether the oligomerization of peroxisomal ABC transporters has any influence on substrate specificity.

### 2.2. Substrate Specificity

Since the cloning of the *ABCD1* gene in 1993 and its association with X-ALD [30], ABCD1 function has been attributed to the transport of saturated and monounsaturated VLCFAs across the peroxisomal membrane for further degradation by β-oxidation. Accumulation of saturated and monounsaturated VLCFAs indeed occurs in the plasma and tissues of X-ALD patients, and is used for diagnosis [31,32]. Due to its importance, studies concerning the structure, function, and defects of ABCD1 have never ceased. Functional complementation experiments in yeast, functional assays in mammalian cells, especially cells coming from X-linked adrenoleukodystrophy (X-ALD) patients, and studies using animal models, mainly knock-out mice, were helpful in clarifying the question of substrate specificity. The *Abcd1* knock-out mice confirmed the human biochemical phenotype, indicating that ABCD1 is indeed involved in the transport of VLCFAs [33,34,35]. Transfection of X-ALD skin fibroblasts with ABCD1 cDNA corrected the β-oxidation defect and restored normal levels of VLCFAs [36,37]. The preference of ABCD1 for saturated FAs was also confirmed in yeast [26,27]. 

Cloned by homology using degenerate primers, the *ABCD2* gene was shown to code for ALDRP, the closest homolog of ALDP [38]. Both proteins display overlapping substrate specificities for saturated and monounsaturated LCFAs and VLCFAs. It explains the correction of β-oxidation defect in X-ALD fibroblasts in case of *ABCD2* overexpression after transfection [39]. Using transgenic expression of *Abcd2* in the *Abcd1* knock-out mouse, Pujol et al. demonstrated that VLCFA accumulation and disease phenotype could be corrected in vivo [40]. This set the basis for a new therapeutic strategy for X-ALD patients aiming at inducing *ABCD2* expression with pharmacological, hormonal, or nutritional management [41,42]. Pharmacological induction of *ABCD2* was indeed shown to compensate for ABCD1 defect in vitro and in rare cases, in vivo, opening the way for clinical trials [43,44,45,46,47,48,49,50,51,52,53,54,55,56,57,58]. 

Functional complementation in yeast model and X-ALD fibroblasts confirmed the functional redundancy for saturated VLCFAs, but also demonstrated the specific role of ABCD2 in PUFA transport, especially DHA and its precursor (C24:6 n-3) [26]. Experiments in mammalian cells confirmed such substrate preference [19,20]. Further studies using the *Abcd2* null mice demonstrated a specific role in MUFA transport, especially for erucic acid (C22:1 n-9) in adipose tissue [59,60] and an extended role in FA homeostasis [61]. 

PMP70, the protein coded by the *ABCD3* gene, was the first identified peroxisomal ABC transporter and is the most abundant peroxisomal membrane protein, at least in hepatocytes [62,63]. Wrongly associated with peroxisome biogenesis [64], ABCD3 is also involved in the transport of various lipids and shows overlapping substrate specificities with ABCD1 when overexpressed [37,65]. Though, ABCD3 clearly has the broadest substrate specificity as it is involved in the transport of LCFAs and VLCFAs but also specifically in the transport of dicarboxylic acids, branched-chain fatty acids, and C27 bile acid intermediates such as di- and tri-hydroxy-cholestanoic acid [65,66,67]. The *Abcd3* knock-out mice indeed revealed a marked accumulation of bile acid intermediates, and ABCD3 was recently associated with a congenital bile acid defect (CBAS5, see below) [67]. Furthermore, a more recent study performed on manipulated HEK-293 cell models proved that ABCD3 is required for the transport of MCFAs across the peroxisomal membrane [68]. 

### 2.3. Mechanism

Conversion of free FAs into CoA esters constitutes an initial activation step before peroxisomal β-oxidation. This reaction is catalyzed by specific acyl-CoA synthetase connected to the cytosolic side of the peroxisomal membrane [69]. It was proved, using protease protection assays, that acyl-CoAs but not free FAs bind to the TMD of the transporter [70]. It is therefore only after activation that the fatty acyl-CoAs are transported to the peroxisomal matrix through peroxisomal ABC transporters. Fatty acyl-CoA are captured on the cytosolic side by the TMD, enhancing the affinity of NBD for ATP. ATP molecules are then hydrolyzed, thus producing the energy needed to switch the conformation of TMD and eventually allowing the translocation of substrates from the cytosol into the peroxisomal matrix [22,71]. However, the exact mechanism of transport remains controversial. Two models are commonly considered. The first implies that esterified FAs are delivered directly to the peroxisomal matrix, whereas in the other model, free FAs are transported into the peroxisomal matrix after the hydrolysis of acyl-CoAs, which are re-esterified by acyl-CoA synthetase when in the peroxisomal lumen. 

Although the process of cleavage and reactivation of acyl-CoAs seems to be a waste of energy as two ATP molecules are needed for the activation reaction, such a mechanism is crucial for the specific permeabilization of the substrates of β-oxidation [18]. Several studies have been done in an attempt to figure out the correct model for the transportation mechanism. Early studies on yeast models have demonstrated that fatty acyl-CoAs are hydrolyzed before being transported. This hydrolysis occurs when acyl-CoAs interacts with the heterodimer Pxa1p-Pxa2p at the cytosolic side of the peroxisomal membrane [72]. The peroxisomal ABC transporters would release a free fatty acid that should be re-esterified inside the peroxisome before its catabolic processing. In addition, intrinsic acyl-CoA thioesterase activity has been found in COMATOSE (CTS), a homolog of human ABCD1 in *Arabidopsis thaliana*, proving again that VLCFA-CoA is hydrolyzed prior to transport [73]. Very recently, the work of Kawaguchi et al. provided further proof of the transport mechanism [74]. After expressing human His-tagged ABCD1 in methylotrophic yeast, they directly demonstrated that ABCD1 transports the FA moiety after the hydrolysis of VLCFA-CoA and that acyl-CoA synthetase is required before the β-oxidation of VLCFA-CoA within the peroxisomes. When it comes to the fate of the free CoA, they are released in the peroxisomal lumen, as revealed using isolated peroxisomes from *Saccharomyces cerevisiae* [75].

Finally, after their re-esterification, substrates are directly delivered to specific acyl-CoA oxidases to initiate the β-oxidation process. Peroxisomal acyl-coenzyme A oxidase 1 (ACOX1) catalyzes the first and rate-limiting step of the β-oxidation pathway dedicated to straight-chain fatty acids, which includes LCFAs, VLCFAs, PUFAs, and dicarboxylic acids [76]. Other acyl-CoA oxidases also exist, ACOX2 and ACOX3. ACOX2 is specific to bile acid intermediates [76] whereas the oxidation of branched-chain FAs depends on both ACOX2 and ACOX3 enzymes [77,78]. Of note, mitochondria catalyze the β-oxidation of the majority of short, medium, and long chain FAs but not that of VLCFAs [79]. In yeast and plants, this process of FA β-oxidation occurs exclusively in peroxisomes, whereas in higher eukaryotes, the catabolism of VLCFAs is initiated solely in the peroxisomes [7,80]. 

## 3. Human Diseases 

### 3.1. X-Linked Adrenoleukodystrophy

X-linked adrenoleukodystrophy (X-ALD, OMIM # 300100) is the most frequent peroxisomal disorder but is still classified as a rare disease, with an estimated incidence of 1:17,000 [81]. Recent therapeutic successes [82,83], and the feasibility and reliability of a diagnosis method based on VLCFA quantification from blood spot [84,85], have prompted some countries to establish systematic screening of newborns. This complex and fatal neurodegenerative disorder is characterized by a huge clinical variability both in the age of onset and in the symptoms [31]. The two main forms are the childhood cerebral ALD (ccALD), characterized by inflammatory demyelination of the central nervous system and the adult form, called adrenomyeloneuropathy (AMN), consisting of a non-inflammatory, slowly progressive demyelination affecting the spinal cord and peripheral nerves. X-ALD is also the main cause of Addison’s disease and adrenal insufficiency may remain the unique symptom of the disease. Since the disease is linked to chromosome X, boys and men are the most severely affected patients. Female carriers usually remain quasi asymptomatic or present only a mild phenotype, but severe forms have also been described [86].

In 1993, using positional cloning, the team of Hugo Moser identified the ABCD1 gene as being responsible for X-ALD [30]. Mutations in the ABCD1 gene have been found in every X-ALD patient and are collected in the X-ALD database (https://adrenoleukodystrophy.info/ accessed on 1 June 2021). In spite of almost 900 non-recurrent mutations, no genotype-phenotype correlation has been described. It is important to note that the majority of missense mutations affect protein stability and result in the absence of the protein. ABCD1 defect results in the accumulation of VLCFAs, mainly C26:0 and C26:1, which accumulate as free FAs or in esterified forms in membrane lipids and cholesteryl esters. This accumulation results from the impossibility of their entry into the peroxisome for their degradation by β-oxidation, but also from an increased endogenous biosynthesis [87]. While the toxicity of VLCFAs has been recognized [88], the sequence of events leading to neurodegeneration and inflammation is still debated. Oxidative stress and cellular components, especially microglial functions, seem to play a major role in the pathogenesis of X-ALD [89,90,91]. 

Therapeutic strategies depend on the clinical symptoms of patients. A majority of X-ALD patients present adrenal insufficiency and require a careful patient follow-up, but hormone-replacement therapy successfully manages the adrenal defect and prevents a potentially fatal Addisonian crisis. Hematopoietic stem cell transplantation (HSCT) has proven efficiency to halt neurological involvement in X-ALD. Since 1990 and the first success of this therapy [92], allogeneic graft has been indicated for boys with ccALD at an early stage of the disease when a compatible donor exists. In 2009, autologous HSCT was demonstrated to be successful to halt cerebral demyelination in two boys with no compatible donors who received their own genetically corrected stem cells [82]. Lentiviral correction of bone marrow derived stem cells and autologous transplantation proved effective in 15 patients in 2017 [83]. These promising results suggest that such a therapeutic strategy may be as effective as allogeneic HSCT. In addition, efforts to find pharmacological strategies targeting oxidative stress, inflammation, or compensatory mechanisms (antioxidant cocktail [93], leriglitazone [94], sobetirome [50,54,55]) are still present. It remains to be evaluated whether such treatments would be useful per se or in combination with HSCT strategies, at least to delay the onset of neurological concerns and permit a lengthening of the time window to allow transplantation.

### 3.2. Congenital Bile Acid Synthesis Defect Type 5

Although mutations were found in the ABCD3 gene of a Zellweger patient [64], further evidence showed that ABCD3 has no link with peroxisomal biogenesis and is definitively not associated with Zellweger Syndrome [95]. ABCD3, which presents partial functional redundancy with ABCD1, has been shown to transport branched-chain FAs, dicarboxylic acids, and bile acid precursors. A few years ago, the accumulation of peroxisomal C27-bile acid intermediates DHCA and THCA, as well as VLCFAs, was described in a young Turkish girl whose parents were consanguineous [67]. The patient presented hepatosplenomegaly and a severe progressive liver disease and she died of complications after liver transplantation. Patient fibroblasts showed reduced numbers of enlarged peroxisomes, as well as reduced β-oxidation of pristanic acid, compared to controls. Immunofluorescence confirmed the absence of ABCD3 in the peroxisomal membrane. A homozygous truncating mutation was identified in the ABCD3 gene of the patient, and the disease was named congenital bile acid synthesis defect (CBAS) type 5 (OMIM # 616278). It should be noted that CBAS type 1, 2, 3, 4, and 6 are associated with mutations in HSD3B7, AKR1D1, CYP7B1, AMACR, and ACOX2 respectively. These genes control key reactions in bile acid synthesis and all the CBAS forms present an autosomal recessive inheritance.

### 3.3. Peroxisomal ABC Transporters and Cancer

Beyond its recognized role in metabolism and redox homeostasis, the peroxisome is now increasingly regarded as a signaling platform and a key organelle in cellular metabolic reprogramming with major consequences on the immune response, cell cycle, and cell differentiation [12,96]. Elegantly presented in the state of the art of Hlaváč and Souček, several studies have revealed a significant association between the level of expression of peroxisomal ABC transporters and various cancers [97]. This suggests a role of these ABC transporters in cell cycle control, cell differentiation, and tumorigenesis. Downregulation of peroxisomal ABC transporters has been observed in several cases: *ABCD1* in melanoma [98] and renal cell carcinoma [99], *ABCD2* in breast cancer [100], and *ABCD3* in ovarian cancer [101] and colorectal cancer [102]. Moreover, a lower prognostic value has been associated with low expression of *ABCD1* in ovarian cancer [103] and low expression of *ABCD3* in colorectal cancer [102]. On the contrary, *ABCD1* and *ABCD3* were found upregulated in breast carcinoma [104], and a positive correlation was observed between *ABCD3* expression and glioma tumor grades [105]. Recently, VLCFA accumulation was associated with colorectal cancer [106]. Increased endogenous elongation appears to be primarily responsible for this observation, but peroxisomal ABC transporters are also likely involved, and the ability to regulate their expression could potentially represent a therapeutic interest in such cancers. Altogether, further studies are required to understand the link between the transport function and metabolic role of peroxisomal ABC transporters and the control of cell cycle with regard to the complexity of tumor heterogeneity.

## 4. Cell, Plant, and Animal Models

Phylogenetic analysis of peroxisomal ABC transporters in eukaryotes shows strong conservation, highlighting their fundamental and specific role in the cellular functions. Interestingly, their substrate specificity seems to become more restrictive with the complexification of the biological systems. Although the number of peroxisomal ABC transporters and their specific functions vary between species, each model of study is of scientific interest and has contributed significantly to the knowledge of peroxisomal ABC transporters. Here are described the main eukaryote models. 

### 4.1. Yeast

In addition to its convenience for genomic modification, *Saccharomyces cerevisiae* is a particularly interesting model for studying the peroxisomal metabolism of lipids, since it can use FAs as its only carbon source and β-oxidation of FAs of all length takes place only in peroxisome. The yeast model expresses only two peroxisomal ABC transporters, called Pxa1p and Pxa2p, which function as a strict heterodimer to import fatty acyl-CoAs into the peroxisomal matrix [107,108,109]. Functional assays and functional complementation experiments of pxa1/pxa2Δ yeast mutants with mammalian peroxisomal ABC transporters were particularly important in studying their transport mechanism and substrate specificity [26,65,75].

### 4.2. Plant

In *Arabidopsis thaliana*, CTS, the human ABCD1 ortholog, is an integral peroxisomal membrane protein composed of two fused half-size transporters. CTS is involved in the import of FAs and phytohormone precursors into the peroxisome where they are β-oxidized [110,111]. The products of this oxidation are involved in the transition from dormancy to germination, root growth, seedling establishment, and fertility [112]. Expression of human *ABCD1* in *A. thaliana* CTS mutant cannot restore the germination and establishment, whereas human *ABCD2* only restores the germination phenotype [113]. These results are related to the physiological differences between plants and mammals, and highlight the differences in substrate specificity between ABCD1 and ABCD2. The plant model was also very important as it showed for the first time the existence of CTS in high molecular weight complexes and allowed the study of the transport mechanism, especially the role of its thioesterase activity [73].

### 4.3. Nematode

*Caenorhabditis elegans* is a well-known worm model in neurobiology studies, but the interest of this model in the field of X-ALD has been shown only very recently. *PMP-4* is one of the five putative peroxisomal ABC transporters identified in *C. elegans* and is the ortholog of human *ABCD1* and *ABCD2*. It is mainly expressed in gut and hypodermis, the main fat storage tissues in the *C. elegans*. Moreover, hypodermal cells have similarities with vertebrate glial cells and participate in neuronal migration [114]. *PMP-4* deficient worms have a normal growth and maturation but show several hallmarks of X-ALD (global VLCFA accumulation, redox imbalance, axonal damage, motility alteration) [115]. Interestingly, the number and the size of lipid droplets (LDs) are increased and can be normalized using a mitochondrial targeted antioxidant. *C. elegans* is therefore a valuable model to study the involvement of FA accumulation and oxidative stress in the pathogenesis of X-ALD but has some limitations since its nervous system is not myelinated. 

### 4.4. Insect

An X-ALD fly model has been generated in *Drosophila melanogaster* using RNA interfering of *dABCD*, the ortholog of *ABCD1*. These flies survive to adulthood but exhibit a specific brain neurodegenerative phenotype with retinal defects including holes and loss of pigment cells associated with death of neurons and glia [116]. Interestingly, cellular targeted disruption of *dABCD* in neurons, but not in glia, triggers the retinal defects. The phenotype is indistinguishable from the one observed in *bgm* (bubblegum) and *dbb* (double-bubble) deficient flies [117]. Both *bgm* and *dbb* genes code for long/very-long-chain acyl-CoA synthetases. The shared neurodegenerative features in *dABCD* and *bgm*/*dbb* deficient flies show that the lipid metabolic pathway is a key component of the X-ALD-like neurodegenerative disease in *Drosophila*. More specifically, experiments achieved with *bgm* and *dbb* deficient flies indicate that the loss of metabolites is the cause of neurodegenerative disease rather than accumulation of substrates (V/LCFAs), as was commonly thought. 

### 4.5. Fish

Zebrafish (*Danio rerio*) has recently been proved to be a useful model for studying the pathogenesis of X-ALD. Indeed, *Abcd1* (the zebrafish ortholog of *ABCD1*), is expressed during development in spinal cord and in the central nervous system especially in the oligodendrocytes and motor neuron precursors, but also in the interrenal gland (functional equivalent of the adrenal cortex) [118]. Zebrafish *Abcd1* mutant models show key biochemical and nervous system alteration features of X-ALD (increased level of C26:0, accumulation of cholesterol, hypomyelinated spinal cord, modified development of interrenal gland and brain, early alteration of motor behavior, decreased survival, and modified oligodendrocytes pattern associated with apoptosis). Interestingly, the motor alteration and the oligodendrocytes pattern can be corrected by human *ABCD1* expression. Moreover, a recent drug screening study showed that chloroquine can improve motor activity in zebrafish *Abcd1* mutant and reduce saturated VLCFA levels [119].

### 4.6. Rat and Mouse

Various cellular models have been created in rodent species to study the function of peroxisomal ABC transporters and the consequences of their defect. Considering that the liver is a platform for peroxisomal lipid metabolism in mammals, the hepatic H4IIEC3 cell line was used to create a specific cell model allowing the inducible expression of a normal or mutated rat Abcd2 protein fused to green fluorescent protein [120]. It allowed to precise the substrate specificity of Abcd2 as well as its dimeric status, and even, to demonstrate for the first time its supradimeric structure. [19,20,28]. To better understand the role of peroxisomal ABC transporters in the glial cells, models of ALD astrocytes have been developed. Astrocytes are known to regulate the inflammatory response. In neurodegenerative diseases, reactive astrocytes secrete inflammatory cytokines, which allow the permeability of the blood-brain barrier (BBB) to peripheral infiltrating immune cells. When *Abcd1* and/or *Abcd2* genes are silenced in mouse primary astrocytes, X-ALD biochemical hallmarks are present (decreased C24:0 β-oxidation, increased C26:0 level), but so are redox imbalance and pro-inflammatory features (increased cytokines expression and nitric oxide production) [121]. These characteristics are inverted by treatment with Lorenzo oil and increased by a long-term VLCFA treatment showing the link between VLCFA accumulation and the pro-inflammatory response of these glial cells [122]. These first results obtained in primary astrocytes led to the development of an immortalized astrocyte cell line [123]. This model should be very useful for studying the mechanisms of astrocyte activation and was used to screen therapeutic compounds such as SAHA, an HDAC inhibitor that normalizes ROS production as well as iNOS and TNF expression [53].

Microglia is also considered a major player in the X-ALD pathogenesis, especially in the inflammatory process. To proceed further, *Abcd1* and/or *Abcd2* deficient microglia cell lines have been obtained using CRISPR/Cas9 gene editing in the mouse BV-2 cell line [124]. The *Abcd1*^−/−^*Abcd2*^−/−^ cells, generated to avoid masking effects due to functional redundancy, show classical X-ALD biochemical hallmarks (increased levels of saturated and monounsaturated VLCFAs) but also increased levels of some LCFAs and PUFAs. Like in brain macrophages from X-ALD patients [125], whorled lipid inclusions, probably corresponding to cholesterol esters of VLCFAs, were observed, making these cells particularly interesting for modelling the human disease. Further studies using these cell lines, alone or in co-culture with glial and/or neuronal cells, should bring new insights for understanding the impact of *Abcd1*/*Abcd2* deficiencies in the microglial function, and could be used for the screening of pharmaceutical compounds useful to halt chronic inflammation in the brains of cALD patients.

In order to study the function of peroxisomal ABC transporters and the pathogenesis of X-ALD in integrated mammalian models, *Abcd1*-, *Abcd2*-, and *Abcd3*-deficient mouse models have been generated [33,34,35,40,67,126]. The *Abcd1* knock-out mice show key biochemical features of X-ALD but develop a late onset progressive neurodegenerative phenotype involving the spinal cord and sciatic nerves without brain damage [127]. In the spinal cord, inflammation is observed in old mice and includes microglia and astrocyte activation [40]. However, microglia activation seems to occur early, probably from eight months of age [91]. VLCFA excess would induce an early oxidative stress leading to mitochondria structural and functional damages as well as an ER stress concomitant with autophagy disruption [128,129,130,131,132]. Although no cerebral phenotype is observed, *Abcd1* knock-out mice can be considered a physiological model of AMN or female myelopathy and can be useful for screening pharmaceutical compounds. Several molecules have thus been tested and have demonstrated their efficacy, including antioxidant compounds that have been proven to reverse oxidative stress in vitro and reduce locomotor impairment [133,134,135]. These hopeful results led to a prospective phase II pilot study that was carried out for 13 AMN patients treated with a cocktail of antioxidant molecules [93]. The study showed that biomarkers of oxidative damage and inflammation were normalized and that patients’ locomotion was improved, paving the way for a hopeful Phase III study. 

Even if the mouse model is attractive because of its phylogenic proximity to humans, it doesn’t reproduce the human brain phenotype of X-ALD. One possible explanation could be related to species and cell-type differences in the expression levels of *ABCD1–3* and functional redundancy issues. Sustaining this hypothesis, a transcriptomic analysis showed that *ABCD2* is not expressed in human microglia and *ABCD3* is 1.6-fold more expressed than *ABCD1* [136], whereas in mouse BV-2 microglial cells, *Abcd2* is 2.5-fold more expressed than *Abcd1* and *Abcd3* is 1.6-fold more expressed than *Abcd1* [124]. In addition, the biochemical and neurological defects observed in the *Abcd1* knock-out mice can be corrected by ubiquitous transgenic expression of *Abcd2* [40]. On the contrary, *Abcd1/Abcd2* double knock-out mice have an earlier and more severe neurological phenotype associated with inflammatory T lymphocyte infiltration in the spinal cord [40]. The *Abcd2* knock-out mice also develop progressive motor disabilities specifically involving sensitive peripheral neurons and spinal cord dorsal and ventral columns and share subcellular abnormalities with the *Abcd*1 knock-out mice (axonal degeneration, C26:0 accumulation, oxidative stress, organelle abnormalities concerning mitochondria, lysosome, endoplasmic reticulum, and Golgi apparatus). This model also revealed the key role of *Abcd2* in adrenals [137] and in adipose tissue and lipid physiology [59,60,61].

In contrast to the *Abcd1* and *Abcd2* knock-out models, the *Abcd3* knock-out mice do not develop peripheral or central neurodegeneration (like *ABCD3* deficiency in humans), but exhibit hepatomegaly associated with abnormalities in peroxisomal FA metabolism, which seems to represent a suitable model for CBAS5 [67].

### 4.7. Human

X-ALD patient skin fibroblasts have, for several years, constituted one of the rare in vitro models of the disease. In 1980, Moser et al. demonstrated for the first time that the accumulation of VLCFAs observed in the brain and adrenals of patients is also present in primary fibroblasts, thus validating this model for X-ALD studies, at least at the biochemical level [138]. Since then, this cellular model has become a platform for a broad variety of analyses concerning lipid metabolism, X-ALD diagnosis, functional characterization of peroxisomal ABC transporters, cellular consequences of *ABCD1* deficiency, and screening of therapeutic compounds. Great scientific advances have emerged from this handy model, but its skin origin is a limitation in pathogenesis studies. Indeed, the gene regulation and function in skin fibroblasts are very far from those of neural, glial and microglial cells. 

The involvement of peripheral blood mononuclear cells (PBMCs) in the inflammation feature of X-ALD was early suspected, since PBMCs from X-ALD patients produce higher levels of inflammatory cytokines than control ones [139,140]. Used in gene therapy, the CD34+ PBMCs (lymphoid and myeloid progenitors) transduced with normal *ABCD1* can efficiently correct the clinical phenotype of the X-ALD patients [82]. Moreover, AMN monocytes have a pro-inflammatory expression pattern and, after differentiation into macrophages, are not able to switch to an anti-inflammatory regenerative state [141]. *Abcd2*, whose expression level is extremely low in these cells, could be a therapeutic target [142]. Therefore, human monocytes can be used to study the inflammatory process and identify compounds capable of inducing *ABCD2* expression, correcting VLCFA level, β-oxidation, and inflammatory features [44,58]. 

The development of the iPSC (induced pluripotent stem cell) technology offers the opportunity to study disease-involved cells with a chosen mutation and a phenotype matching physiology. Several iPSC models have successfully been obtained from skin fibroblasts of cALD and AMN patients [143,144,145,146,147]. Gene expression profiling shows that X-ALD iPSCs have differentially expressed genes compared to control iPSCs, among which some are positively correlated to the severity of the disease (cALD versus AMN) [148]. When iPSCs are differentiated into oligodendrocytes or astrocytes, the VLCFA level is increased and is higher in cALD differentiated cells than in AMN cells, whereas no VLCFA accumulation is observed in neurons [144]. iPSC-derived astrocytes show pro-inflammatory features that also correlate with the severity of the phenotype. The differentiation of microglia from iPSC also seems to be a promising model, as differentiated microglia show the main phenotype of primary fetal and adult human microglia including phagocytic and inflammatory capacity [146]. In addition, cALD iPSCs differentiated in brain microvascular endothelial cells show impaired BBB function as well as lipid metabolism modifications and interferon activation [149], and could lead to the study of an important factor of brain pathogenesis in X-ALD. Altogether, these works show that iPSC-derived brain cells should allow the study of the pathogenesis of X-ALD in detail, permit the identification of biomarkers, and screen new therapeutic molecules. Co-culture experiments are expected to provide new insight into intercellular communication in the brain. 

In conclusion, for forty years, enormous progress has been made in the knowledge of peroxisomal ABC transporters thanks to the development and the use of cell and animal models. If no model exactly mimics the human X-ALD, there is no doubt that the new technological developments will offer opportunities to progress in the study of the role of peroxisomal ABC transporters in the neuronal, glial, and microglial intercellular communications. 

## 5. Protein Interactions and Unexpected Roles

Physical interaction between peroxisomal ABC transporters and other proteins have been reported in several studies. Most binding partners are involved in lipid metabolism. Here, we propose to review these binding partners for which strong interaction experiments have been obtained, or for which further investigations are needed to be reliable. 

Peroxisomal membrane insertion, substrate binding, transport mechanism, and the potential novel functions of peroxisomal ABC transporters require protein interactions. Since their first identification, many efforts have been developed to understand how peroxisomal ABC transporters are targeted to the peroxisomal membrane. PEX19p, a cytosolic peroxin, was identified as an interactor of ABCD1, ABCD2, and ABCD3 by using the yeast two-hybrid system and in vitro GST pull-down assays [150]. In addition to being involved (in association with PEX3) in the correct peroxisomal targeting of peroxisomal membrane proteins (PMPs), PEX19p may also function as a protein chaperone to prevent aggregation of newly synthesized PMPs [151]. It’s worth noting that PEX19p is the only ABCD2 binding partner that has been reported in the literature, probably due to the fact that ABCD2 is much less closely studied than ABCD1 and ABCD3 since it is not responsible for a genetic disease when mutated. To identify potential ABCD2 binding partners, we used the inducible H4IIEC3 cell model, which expresses ABCD2-EGFP depending on the presence of doxycycline [28]. We performed quantitative ABCD2 co-immunoprecipitation assays coupled with tandem mass spectrometry. Differential analysis between cell samples was done to limit detection of false-positive interactions. The list of potential binding partners of ABCD2 is given in Table 1 and includes 13 non-redundant proteins exclusively detected in the positive samples [28]. Only one subunit of the oligosaccharyl transferase (OST) complex that catalyzes the N-glycosylation of newly translated proteins in the endoplasmic reticulum was identified as a potential ABCD2 binding partner: the dolichyl-diphosphooligosaccharide protein glycosyltransferase subunit 2 (RPN2) (Table 1). On the other hand, RPN2 has also been identified by proteomic analyses in a subclass of peroxisome expressing ABCD2 [152]. These data are still quite surprising since peroxisomal ABC transporters, such as most PMPs, are known to be synthesized on free polysomes and to further insert directly from the cytosol into the peroxisomal membrane. It is worth noting that an indirect peroxisomal targeting pathway exists via the ER since several PMPs are found glycosylated [153]. The potential interaction of ABCD2 with the OST complex involved in N-glycosylation is inconsistent with the absence of routing through the ER with respect to peroxisomal ABC transporters. Nevertheless, proteomic data leading to identification is not robust since the protein probability for RPN2 is low (0.7224) (Table 1). 

Besides the question of routing and peroxisomal targeting, the main putative ABCD2 partners revealed in this study were associated with lipid metabolism. Unsurprisingly, several binding partners identified have a role in FA activation, which is required on both sides of the peroxisomal membrane. At the cytoplasmic side of the peroxisomal membrane, a complex FA synthesis-transport machinery was evidenced by using a multi-approach method, combining GST pulldown experiments, mass spectrometry (LC/MS), co-immunoprecipitation assays, and bioluminescence resonance energy transfer (BRET) measurements [154]. This machinery consists of the binary interaction of ABCD1/3 with proteins carrying functions associated with FA activation/transport (ACSVL4) and FA synthesis (ACLY, ATP citrate lyase; FASN, FA synthase). On the inner surface of the peroxisomal membrane, studies using a yeast two hybrid system and surface plasmon resonance techniques indicate that the very long-chain acyl-CoA synthetase 1 (ACSVL1) interacts with ABCD1 [155]. In *Saccharomyces cerevisiae*, peroxisomal ABC transporters (Pxa1p and Pxa2p) functionally interact with the acyl-CoA synthetase Faa2p on the inner surface of the peroxisomal membrane for subsequent re-esterification of the VLCFAs [72]. In this model, whether or not a physical interaction with acyl-CoA synthetases exists remains to be investigated. In *Arabidopsis thaliana*, peroxisomal long-chain acyl-CoA synthetases (lacs6 and lacs7) physically and functionally interact with CTS, as assessed by co-immunoprecipitation experiments [73]. 

In our study aiming at identifying ABCD2 binding partners, the fatty-acid amide hydrolase 1 (FAAH1) exhibited the highest fold change (Table 1, FC = 5.02). This endoplasmic reticulum enzyme is the main enzyme involved in anandamide hydrolysis and plays an important role in endocannabinoid metabolism degrading the FA amides to the corresponding fatty acids, with a PUFA preference over MUFAs and saturated fatty acids [156,157]. Interestingly, FAAH1 catalyzes the conversion of the ethanolamine amide form of DHA (N-docosahexaenoyl ethanolamine) to DHA [158]. The interaction of FAAH1 with ABCD2 could be consistent with the role of FAAH1 as a supplier of ABCD2 substrates (DHA and other PUFAs) for further degradation in the peroxisome by β-oxidation. 

Concerning ether lipid biosynthesis, the peroxisomal enzyme alkyl-dihydroxyacetone phosphate synthase (AGPS) is suggested to interact with ABCD1, as assessed by an integrative global proteomic profiling approach based on chromatographic separation [159]. Ether lipid biosynthesis starts in the peroxisome with the transfer of the acyl group of fatty acyl-CoAs to dihydroxyacetonephosphate (DHAP), generating an acyl-DHAP. The second peroxisomal step is catalyzed by AGPS, which exchanges the acyl chain for an alkyl group, yielding an alkyl-DHAP. After a final peroxisomal step, the ether lipid biosynthesis is completed in the ER. This global proteomic analysis showed that AGPS failed to interact with ABCD2, just as our co-immunoprecipitation coupled to proteomic analysis [28].

β-oxidation of MCFAs to LCFAs mainly takes place in the mitochondria, whereas VLCFAs are first metabolized down to octanoyl-CoA in the peroxisome for further degradation in the mitochondria. Surprisingly, proteomic data supported by co-immunoprecipitation experiments evidenced a physical interaction between a long-chain acyl-CoA synthetase 1 (ACSL1) localized in the ER and ABCD3 [159]. This could be in agreement with the role of ABCD3 in the β-oxidation of lauric and palmitic acids [68]. In addition, ACSL1 has been shown to interact with ACBD5 [160], a peroxisomal membrane protein suggested to function as a membrane-bound receptor for VLCFA-CoA in the cytosol to bring them to ABCD1 [161]. Whether ACSL1 transfers other unidentified lipid species to ACBD5, ABCD1, or ABCD3 for peroxisomal degradation needs further investigation.

Other potential binding partners of ABCD2 identified are involved in mitochondrial FA metabolism, such as the carbonyl reductase family member 4 (CBR4), the long-chain acyl-CoA synthetase 3 (ACSL3), and the long-chain acyl-CoA synthetase 5 (ACSL5) (Table 1). These enzymes were identified with less confidence (fold change <2). Although linked to lipid metabolism, CBR4 is a matrix mitochondrial enzyme. ACSL3 and ACSL5 do not activate VLCFAs, nor does ACSL1, which nevertheless has been found to interact with ABCD3 [160] as discussed above. 

Peroxisomes contain enzymes involved in the α-oxidation of phytanic acid. Large-scale mapping of protein–protein interactions by mass spectrometry identified a single interaction between peroxisomal proteins i.e., the peroxisome matrix phytanoyl-CoA 2-hydroxylase (PHYH) and ABCD3 [162]. This interaction makes sense since, after activation of phytanic acid, phytanoyl-CoA is imported into the peroxisome by ABCD3 and enters in the peroxisomal α-oxidation pathway of which PHYH is the first enzyme (Figure 1).

The recent demonstration of ABCD1 interaction with M1 spastin, a membrane-bound AAA ATPase found on LDs, suggests the involvement of ABCD1 in inter-organelle FA trafficking [163]. Actually, ABCD1 forms a tethering complex with M1 spastin as assessed by co-immunoprecipitation experiments to connect LDs to peroxisomes. Furthermore, by recruiting IST1 and CHMP1B to LDs, M1 spastin facilitates LD-to-peroxisome FA trafficking. Whether M1 spastin-ABCD1 interaction directly promotes fatty acids channeling into peroxisomes remains unclear. It is worth noting that among proteins detected in our ABCD2 interactome study, the spectrin alpha chain, non-erythrocytic 1 (SPTN1) was identified as a potential ABCD2 binding partner (Table 1). As a cytoskeletal protein, SPTN1 is known to be involved in stabilization of the plasma membrane and to organize intracellular organelles [164]. These data corroborate the existence of peroxisome interconnection with LDs and the cytoskeleton [165,166].

Related to calcium signaling, the sarcoplasmic/endoplasmic reticulum calcium ATPase 2 (AT2A2) was identified with a high fold change (FC = 4.71), which ensures a specific interaction with ABCD2 (Table 1). Besides, its homolog (ATPase1) has been identified as well by proteomic analyses in a subclass of peroxisome expressing ABCD2 [152]. AT2A2 transfers Ca2+ from the cytosol to the ER and is then involved in calcium signaling. Coincidently, disturbed calcium signaling was suggested to be associated with the pathogenesis of X-ALD [122]. Involved in maintaining intracellular calcium homeostasis, the plasma membrane calcium-transporting ATPase 1 (AT2B1) was identified, though with less confidence (fold change <2). Actually, its physical interaction with ABCD2 remains questionable since it is expressed at the plasma membrane. Nevertheless, several high throughput studies using robust affinity purification-mass spectrometry methodologies to elucidate protein interaction networks have revealed the interaction of ABCD1 with AT2B2 [167] and ABCD3 with AT2B2 and AT2A2 [168,169]. Hence, clusters of arguments indicate that peroxisomal ABC transporters could be linked to calcium signaling, but deciphering molecular interaction networks would be required to confirm this hypothesis.

Identification in the putative ABCD2 partners of the serum paraoxonase/arylesterase 1 (PON1), an antioxidant enzyme synthetized and secreted by the liver in the serum [170] where it is closely associated with high density lipoprotein (HDL), could be at first glance intriguing (Table 1). Nevertheless, in the liver, PON1 is primarily localized in microsomal fraction where the enzyme is associated with vesicles derived from the ER [171]. The potential intracellular interaction with ABCD2 remains to be elucidated. Noteworthy, PON1 activity and polymorphisms have been associated with neurodegenerative diseases [172], of which X-ALD is not evoked.

The binding partners of peroxisomal ABC transporters discussed in this review are mainly linked to lipid metabolism (PUFA metabolism, α-oxidation pathway, and ether lipid biosynthesis) and are consequently found in the cytosol, in the peroxisomal membrane, or in the peroxisomal matrix. However, binding partners were identified in other cell compartments. Since peroxisomal lipid metabolism requires cooperation and interaction with mitochondria, ER and LDs, peroxisomal ABC transporters, through their interactome, could therefore actively participate in this intracellular metabolic network. Peroxisome-organelle interactions have physiological relevance [166,173], and peroxisomes are increasingly considered important intracellular signaling platforms that modulate physiological processes such as inflammation, innate immunity and cell fate decision [12,174,175]. Peroxisomal ABC transporters would play an essential part in this emerging role of peroxisomes in signaling pathways such as calcium signaling as highlighted in this review.

## 6. Conclusions

Transcriptomic, proteomic, and lipidomic studies, which have multiplied in the last few years, have confirmed and/or revealed the involvement of peroxisomal metabolism in various biological processes essential for cellular adaptation, brain homeostasis, or even immune response and inflammation. Peroxisomal ABC transporters constitute a pathway for the entry of various lipid substrates into the peroxisome mainly for their degradation but also for the synthesis of bioactive lipids impacting membranes and signaling pathways. It is therefore quite logical that the role of peroxisomal ABC transporters is now extended to unexpected biological processes. Since their cloning in the 90s, the lack of good antibodies, the rarity of relevant cell models, the fragility of the peroxisomal membrane, and other difficulties have constituted a real handicap towards performing functional assays and in vitro transport reconstitutions, and progressing in the understanding of the role of peroxisomal ABC transporters. The emergence of new cell models and the rise of model organisms, as well as cell reprogramming and CRISPR gene editing technologies, suggest that major new discoveries will be made soon that reveal their role in physiological and pathological situations.

## Figures and Tables

**Table 1:** List of proteins identified in co-immunoprecipitated ABCD2-EGFP complex by liquid chromatography coupled with tandem mass spectrometry (modified from [28]).

Protein Accession	Protein Name		Protein Probability	Fold Change ^a^
Q9QY44	ABCD2	ATP-binding cassette sub-family D member 2	1	12.42
P97612	FAAH1	Fatty-acid amide hydrolase 1	1	5.02
P11507	AT2A2	Sarcoplasmic/endoplasmic reticulum calcium ATPase 2	1	4.71
P07340	AT1B1	Sodium/potassium-transporting ATPase subunit beta	1	2.48
P55159	PON1	Serum paraoxonase/arylesterase 1	1	2.32
D3ZHR2	ABCD1	ATP-binding cassette sub-family D member 1	1	<2
P16970	ABCD3	ATP-binding cassette sub-family D member 3	1	<2
Q7TS56	CBR4	Carbonyl reductase family member 4	1	<2
P11505	AT2B1	Plasma membrane calcium-transporting ATPase 1	1	<2
P16086	SPTN1	Spectrin alpha chain, non-erythrocytic 1	1	<2
Q63151	ACSL3	Long-chain acyl-CoA synthetase 3	0.9997	<2
O88813	ACSL5	Long-chain acyl-CoA synthetase 5	0.9994	<2
P14408	FUMH	Fumarate hydratase, mitochondrial	0.8013	<2
P25235	RPN2	Dolichyl-diphosphooligosaccharide—protein glycosyltransferase subunit 2	0.7224	<2

^a^ Statistical significance was obtained for proteins identified with a fold-change >2.

## Abbreviations

ABC	ATP-binding cassette
ACOX1	Acyl-coenzyme A oxidase 1
AMN	Adrenomyeloneuropathy
BBB	Blood-brain barrier
cALD	Cerebral adrenoleukodystrophy
CBAS	Congenital bile acid synthesis defect
CNS	Central nervous system
CoA	Coenzyme A
CTS	Comatose
DHA	Docosahexaenoic acid, C22:6 n-3
ER	Endoplasmic reticulum
FA	Fatty acid
FC	Fold change
HSCT	Hematopoietic stem cell transplantation
iPSC	Induced pluripotent stem cell
LCFA	Long-chain fatty acid
LD	Lipid droplet
MCFA	Medium-chain fatty acid
MUFA	Monounsaturated fatty acid
NBD	Nucleotide binding domain
PBMC	Peripheral blood mononuclear cell
PMP	Peroxisomal membrane protein
PUFA	Polyunsaturated fatty acid
TMD	Transmembrane domain
VLCFA	Very long-chain fatty acid
X-ALD	X-linked adrenoleukodystrophy
